# Effect of Graphene Oxide on the Properties of Poly(3-Hydroxybutyrate-*co*-3-Hydroxyhexanoate)

**DOI:** 10.3390/polym13142233

**Published:** 2021-07-07

**Authors:** Ana M. Díez-Pascual

**Affiliations:** Universidad de Alcalá, Facultad de Ciencias, Departamento de Química Analítica, Química Física e Ingeniería Química, Ctra. Madrid-Barcelona, Km. 33.6, Alcalá de Henares, 28805 Madrid, Spain; am.diez@uah.es; Tel.: +34-918-856-430

**Keywords:** graphene oxide, poly(3-hydroxybutyrate-*co*-3-hydroxyhexanoate), barrier properties, mechanical properties, nanocomposites, food packaging

## Abstract

The main shortcomings of polyhydroxybutyrate (PHB), which is a biodegradable and biocompatible polymer used for biomedical and food packaging applications, are its low thermal stability, poor impact resistance and lack of antibacterial activity. This issue can be improved by blending with other biodegradable polymers such as polyhydroxyhexanoate to form poly(3-hydroxybutyrate-*co*-3-hydroxyhexanoate) (PHBHHx), which is a copolymer with better impact strength and lower melting point. However, PHBHHx shows reduced stiffness than PHB and poorer barrier properties against moisture and gases, which is a drawback for use in the food industry. In this regard, novel biodegradable PHBHHx/graphene oxide (GO) nanocomposites have been prepared via a simple, cheap and environmentally friendly solvent casting method to enhance the mechanical properties and antimicrobial activity. The morphology, mechanical, thermal, barrier and antibacterial properties of the nanocomposites were assessed via several characterization methods to show the enhancement in the biopolymer properties. The stiffness and strength of the biopolymer were enhanced up to 40% and 28%, respectively, related to the strong matrix-nanofiller interfacial adhesion attained via hydrogen bonding interactions. Moreover, the nanocomposites showed superior thermal stability (as far as 40 °C), lower water uptake (up to 70%) and better gas and vapour barrier properties (about 45 and 35% reduction) than neat PHBHHx. They also displayed strong biocide action against Gram positive and Gram negative bacteria. These bio-based nanocomposites with antimicrobial activity offer new perspectives for the replacement of traditional petroleum-based synthetic polymers currently used for food packaging.

## 1. Introduction

**Scheme 1:**
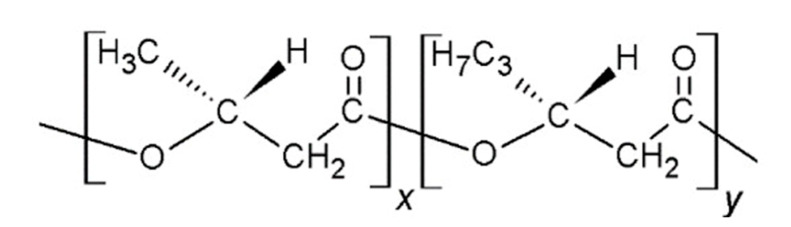
Chemical structure of poly(3-hydroxybutyrate-*co*-3-hydroxyhexanoate) (PHBHHx). “x” and “y” represent the molar fraction of hydroxybutyrate and hydroxyhexanoate units, respectively.

Currently, plastic pollution is a severe threat facing humans, animals and plants. The development of bioplastic materials is imperative in order to solve our global environmental challenges and to preserve the welfare of our world. In this regard, bio-based plastics are pursued since they are expected to replace fossil-based plastics. Amongst the most promising materials being developed is polyhydroxybutyrate (PHB), which is a microbial polyester belonging to the polyhydroxyalkanoate (PHA) family. This biocompatible and non-toxic polymer is biosynthesised by bacterial fermentation from renewable resources such as cane sugar and accumulated by a number of specialised bacterial strains [1]. It is highly crystalline due to its linear chain structure and also has a number of advantages over synthetic polymers for certain biomedical applications and the production of food packaging, including superior barrier performance to both polyethylene (PE) and polypropylene (PP) and it is also more rigid than PP [2]. Moreover, it is biodegradable and the biodegradation takes place within a reasonable timescale when the material is in contact with degrading microorganisms in biologically active environments such as soils, fresh water and so forth. One major challenge of PHB is its narrow processing window, since the degradation and the melting temperatures are too close and, hence, is vulnerable to thermal degradation [3]. Many strategies have been assessed to widen the processability of PHAs. One of the approaches is to blend with other polymers such polylactide acid (PLA) [4] and polycaprolactone (PCL) [5] or reinforcing with nanomaterials including organically modified montmorillonite (OMMT) [6], multi-walled carbon nanotubes (MWCNTs) [7], nano-hydroxyapatite (HA) [8] or zinc oxide (ZnO) [9,10]. In particular, it can be polymerised with 3-hydroxyhexanoate units to yield poly(3-hydroxybutyrate-*co*-3-hydroxyhexanoate) (PHBHHx), with a chemical structure shown in Scheme 1.

This copolymer shows improved processability, higher ductility and better impact strength than other members of the PHA family. However, it displays lower crystallinity and, henceforth, lower stiffness and poorer barrier properties against moisture and gases, which is a shortcoming for its use as food packaging material. In order to increase its range of applications, strategies such as the incorporation of nanofillers [11] or polymer blending [12,13] are pursued. Better mechanical performance and higher thermal and barrier properties have been obtained upon addition of small amounts of nano-sized fillers into this biopolymer matrix, albeit the enhancements are still not sufficient for industrial applications. Moreover, literature studies dealing with PHBHHx nanocomposites are very scarce.

On the other hand, the exceptional physical properties of graphene such as high electrical conductivity, mechanical strength, thermal stability and large surface area render it a potentially ideal filler to enhance the properties of polymeric matrices for diverse applications [14,15]. However, since graphene does not comprise any reactive functional groups, its surface is inert and its interaction with the polymer matrix is weak which hinders its final processing. In order to solve such drawbacks, graphene can be modified via oxidation to yield graphene oxide (GO), which is a derivative that is comprised of oxygenated surface groups, namely epoxy and hydroxyl on the basal planes and carboxylic acids on the edges [16].

GO is a water-soluble nanomaterial and can be easily exfoliated in aqueous media upon sonication. Furthermore, it shows other advantages such as amphiphilicity, biocompatibility [17], high antibacterial activity and the ability to interact with biological cells and tissues. In fact, several articles have reviewed the antimicrobial properties of G-based materials [18,19]. Among the chemical mechanisms of bactericide activity are the oxidative stresses due to excess production of reactive oxygen species (ROS). These are extremely destructive to organisms at high concentrations and causes peroxidation of lipids and oxidation of proteins; and damage to nucleic acids, enzyme inhibition, activation of programmed cell death (PCD) pathway and ultimately results in the death of the cells. Several physical mechanisms, including direct contact of the G sharp edges with the bacterial membrane, wrapping/entrapment of the bacterial cell and lipid extraction, have also been proposed [20,21]. Overall, it is a perfect candidate to enhance the mechanical properties and antibacterial activity of biopolymers for practical applications such as food packaging.

The present article focuses on the preparation and characterisation of novel PHBHHx/GO nanocomposites via simple solution casting. Nanocomposites with different GO concentrations have been prepared and their morphology, crystalline structure, thermal stability, stiffness, strength, toughness, water uptake, oxygen permeability and antibacterial properties have been investigated.

## 2. Materials and Methods

### 2.1. Reagents

Poly(3-hydroxybutyrate-*co*-3-hydroxyhexanoate), PHBHHx, (*M*_n_ = 440,000 g/mol; *T*_g_ = 2 °C, *T*_m_ =110 °C, d_25°C_ = 1.25 g/cm^3^, HHx = 12 mol%) was supplied by Procter and Gamble Co. (Cincinnati, OH, USA). The co-polyester was purified by dissolution in hot chloroform, precipitation in methanol and drying under a vacuum at 70 °C for 24 h. Graphite powder was obtained from Bay Carbon, Inc. (Bay City, MI, USA) and used for the synthesis of graphene oxide (GO). Potassium permanganate, hydrogen peroxide, phosphorus pentoxide, potassium persulfate and 95–98% sulfuric acid were obtained from Sigma-Aldrich (Madrid, Spain) and used as received. 

### 2.2. Synthesis of GO

GO was synthesised via a modified Hummers’ method from graphite powder as reported elsewhere [22]. In short, 5 g of graphite powder was heated with 30 mL of H_2_SO_4_, 5 g of P_2_O_5_ and 5 g of K_2_S_2_O_8_ at 80 °C for 6 h. After cooling, deionised water was added to the mixture and kept under stirring overnight. The product was filtered, dried under air and mixed with 30 g of KMnO_4_, 60 mL of H_2_O_2_ and 200 mL of H_2_SO_4_. The solution was heated to 80 °C in an oil-bath and kept stirring for 24 h. The final product was purified by centrifugation, followed by several cycles of washing with H_2_O_2_/H_2_SO_4_, bath ultrasonication and washed with deionised water. The GO aqueous solution was finally freeze-dried under reduced pressure (0.1 torr, −50 °C). According to elemental analysis, the oxygen content in GO was 51.96%.

### 2.3. Preparation of PHBHHx/GO Nanocomposites

Nanocomposites with GO loadings of 0.5, 1.0, 2.0 and 5.0 wt% were prepared by ultrasonication followed by solution casting. The required amount of GO was dispersed in ethanol by ultrasonication at 200 W for 40 min. Afterward, the PHBHHx was dissolved in the GO dispersion upon heating at 50 °C and the mixture was ultrasonicated for another 20 min. The resulting product was poured into a glass Petri dish and finally dried under vacuum for 24 h to eliminate the residual solvent. Representative photographs of neat PHBHHx and PHBHHx/GO (5.0 wt%) are shown in Figure S1 (Supplementary Materials). PHBHHx is transparent and shows a very smooth surface, while the nanocomposites are more opaque and display higher roughness.

### 2.4. Characterization Techniques 

Scanning electron microscopy (SEM) micrographs were attained with a SU8000 Hitachi scanning electron microscope (Minato-ku, Tokyo, Japan) at an acceleration voltage of 10.0 kV and an emission current of 10 mA. The samples were first cryofractured in liquid nitrogen and then covered with a ∼5 nm Au:Pd overlayer to prevent charge accumulation during electron irradiation. A contact profilometer was used to estimate the average surface roughness of the samples.

The Fourier Transformed Infrared (FT-IR) Spectra were recorded at 25 °C on a PerkinElmer Spectrum One spectrometer (PerkinElmer Inc., Waltham, MA, USA) comprised of an attenuated total reflectance (ATR) sampling accessory (diamond crystal) and a laser excitation source at 632.8 nm. Spectra were recorded with an incident laser power of 1 mW and a resolution of 4 cm^−1^. Four scans were collected for each sample in the wavelength range between 4000 and 400 cm^−1^.

The thermal stability of the composites was analysed by thermogravimetric analysis (TGA) under a nitrogen atmosphere using a TA Instruments Q50 thermobalance (TA Instruments Ltd., Hertfordshire, UK) at a heating rate of 10 °C/min. Prior to the measurements, samples were dried overnight at 50 °C. Experiments were carried out on samples with an average mass of 20 mg, with a purge gas flow rate of 60 mL/min.

Tensile tests under room temperature conditions were performed with an Instron 5565 Testing Machine (Norwood, MA, USA) using a 1 kN load cell and at a crosshead speed of 10 mm/min. Five specimens for each type of nanocomposite were tested to check for repeatability.

In order to calculate the water uptake (WU), samples were dried in a desiccator at 0% relative humidity (RH) for one week. Then, they were placed in a beaker at 100% RH and absorbed water until a constant weight was reached. Water uptake was calculated according to the following equation:

WU (%) = [(*W_f_* − *W_i_*)/*W_i_*] × 100 (1)

where *W_i_* and *W_f_* are the initial and final weight of the samples, respectively. Five replicates for each sample were measured using high-precision analytical balances (±0.001 g) and the average value is reported. 

The water vapour permeability (WVP) was measured at 25 °C following the gravimetric method ASTM E96-95 standard [23] with Payne permeability cups. Samples were equilibrated at 54% RH by using magnesium nitrate-6-hydrate. WVP was calculated according to the following equation:

WVP = (Δ*m* × l)/(*A* × *t* × Δ*P*) (2)

where Δ*m* is the weight loss of each cup, l the film thickness, A the contact area, *t* the time and Δ*P* the partial pressure difference between the inside and outside of the cup. For each sample, WVP measurements were repeated five times and the average value is reported.

The oxygen permeability (OP) was evaluated at 25 °C on films equilibrated at 54% RH by measuring the oxygen transference rate (OTR) with a gas permeability tester following the ASTM D3985-05 standard [24]. OP was calculated as follows:

OP = (OTR × l)/Δ*P* (3)

where l is the average film thickness and Δ*P* the difference between oxygen partial pressure across the film. Tests were performed in quadruplicate and average values and standard errors are provided.

The antibacterial activity of the samples was tested against two types of bacteria: Gram positive Staphylococcus aureus (*S*. *aureus*, ATCC 12600) and Bacillus subtilis (*B. subtilis*, ATCC 23857); and Gram negative Escherichia coli (*E. coli*, ATCC 25922) and Burkholderia cepacia (*B. cepacia*, ATCC 25416). Before the tests, samples were sterilised in an autoclave and subsequently submerged in a nutrient broth of 2.0 × 10^6^ colony-forming units per milliliter (CFU mL^–1^). After an incubation period of 24 h at 37 °C, the number of viable microorganism colonies was counted manually by using a pen and a click-counter and the results were expressed as mean CFU per sample. The antibacterial activity (AA) was calculated by using the following equation:

AA = log(viable cell count_control_ / viable cell count_composite_) (4)

where a beaker containing bacteria and no sample was used as control. Experiments were carried out in triplicate and the average values are reported.

## 3. Results and Discussion

### 3.1. Surface Morphology and Crystalline Structure 

**Figure 1:**
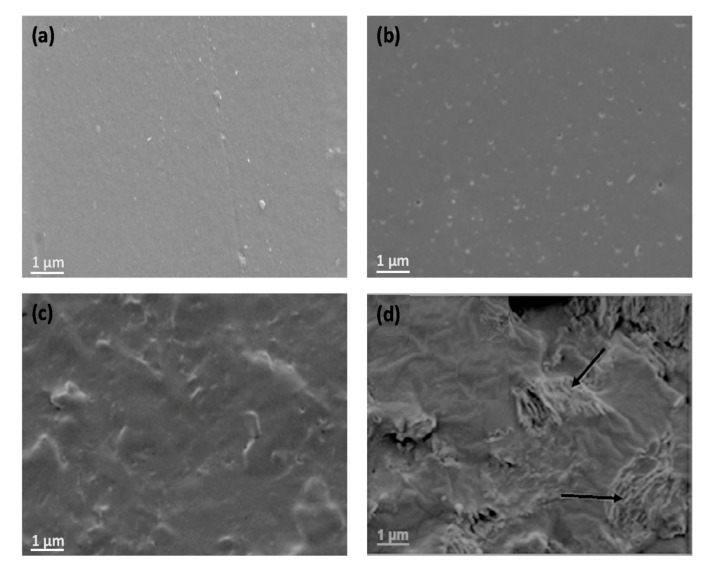
SEM images of PHBHHx (**a**) and the nanocomposites with 0.5 (**b**), 2.0 (**c**) and 5.0 (**d**) wt% graphene oxide (GO) content. The arrows point the GO sheets within the PHBHHx matrix.

Morphology can play a key role in determining the mechanical and barrier properties of polymeric nanocomposites. Thus, SEM analysis was carried out to investigate the morphology and state of dispersion of GO within the biopolymer matrix. Figure 1 presents typical SEM images of PHBHHx/GO nanocomposites. 

The mean surface roughness (R_a_) of neat PHBHHx and the nanocomposites was measured and the results are collected Table 1. Neat PHBHHx (Figure 1a) has a regular and smooth surface due to its relatively low crystallinity [12], as demonstrated by XRD analysis. Small cavities are found on the PHBHHx surface formed during solvent evaporation. Upon increasing GO concentration, the surface becomes rougher by up to 3.5-fold at the highest loading. The increased surface roughness of the nanocomposites compared to PHBHHx could be an advantage for certain applications such as tissue engineering since it favors cell proliferation and differentiation. In the nanocomposites, the GO sheets are found to be homogeneously distributed and well distributed within the matrix, without the occurrence of clusters or agglomerates. They appear as stacked nanosheets with a crumpled and rippled structure (Figure 1d); due to its high specific surface area, GO flakes are prone to interact via π–π interactions, Van der Waals forces and H-bonding. The flakes seem to be bended with a high degree of flexibility. On the other hand, no voids or discontinuities are detected between the GO nanosheets and the polymer, indicating good compatibility between the two phases. 

**Figure 2:**
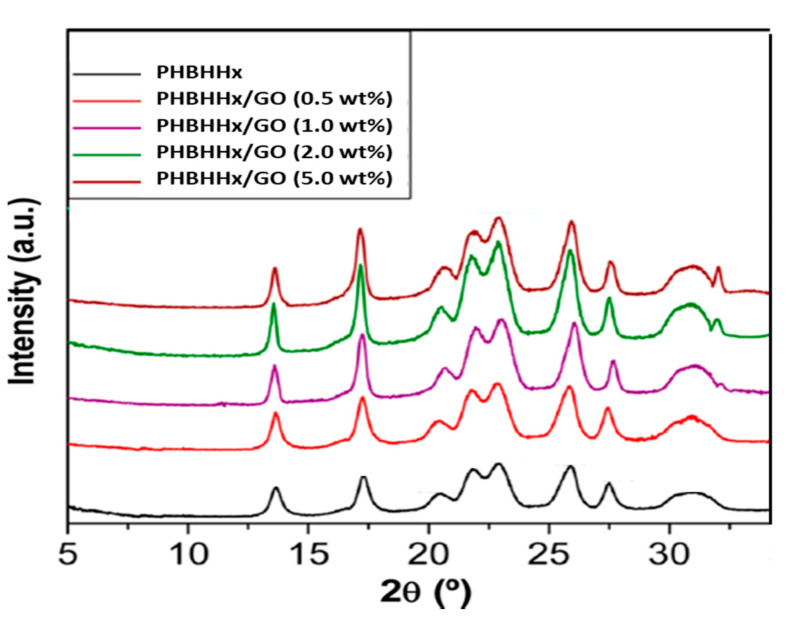
XRD patterns of PHBHHx and the nanocomposites with different GO contents.

The histograms of the thickness distribution obtained from GO nanosheets are shown in Figure S2 (Supplementary Materials). As can be inferred from the statistics, the distribution of the flake thickness of GO is unimodal in the range of 20−100 nm, with a weighted mean value of 65 nm. Overall, SEM analysis corroborates that the ultrasonication step was indeed effective to disrupt the aggregation of GO nanosheets and thus increases the surface area available for interacting with the biopolymer, which further results in a good dispersion of the nanomaterial without the need for surface functionalisation treatments or compatilibising agents; this renders the fabrication process of these nanocomposites simple, cheap and environmentally friendly. In order to obtain further information about the effect of GO onto the crystalline structure of PHBHHx, XRD analysis was performed and the patterns for the different samples are shown in Figure 2. 

Neat PHBHHx displays peaks at 2θ values of 13.7, 17.3, 20.3, 21.6, 22.8, 25.7, 27.5 and 30.9°, which matches the diffraction of the (020), (110), (021), (101), (111), (121), (040) and (200) crystalline planes, respectively. This is essentially the same structure as that of the homopolymer, in which the unit cell is orthorhombic with a space group P2_1_2_1_2_1_-D2 [25]. The same peaks can be observed in the diffractograms of the nanocomposites, indicating that the crystalline structure of the matrix is preserved. Nonetheless, the nanocomposites display much shaper peaks (narrower and with increased height), which is indicative of higher crystallinity. Thus, GO can act a as nucleating agent for the matrix crystallization, resulting in the formation of bigger and more perfect crystals, as previously reported for other nanofillers such as ZnO or boron nitride [9,25]. Indeed, the superior nucleating efficiency of GO has been previously reported for other biodegradable polymeric matrices such as poly (ethylene glycol) (PEG) related to the large nanofiller surface area [26]. In particular, for the nanocomposite with 2.0 wt% GO, about 25% decrease in the full width at half-maximum (FWHM) of the (110) peak is found, which is indicative of a larger crystallite size since the peak width is inversely proportional to the crystallite size according to the Scherrer formula [27].

### 3.2. FT-IR Study

**Figure 3:**
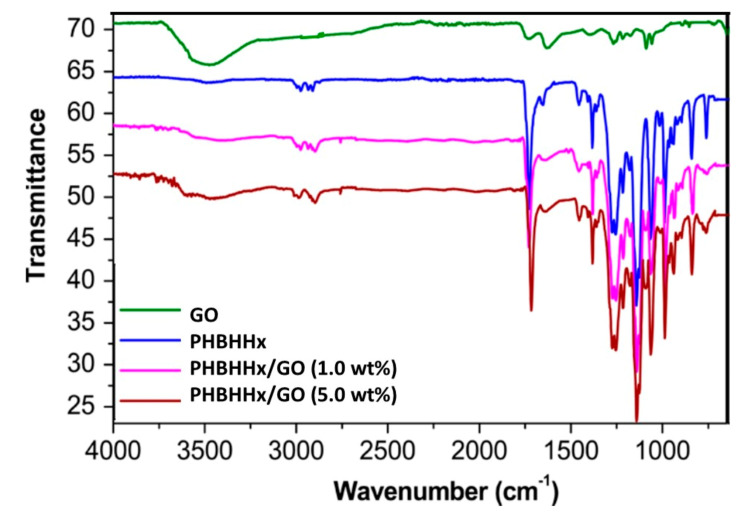
FTIR spectra of PHBHHx, GO and the nanocomposites with 1.0 and 5.0 wt% loading.

The FTIR spectra of GO, PHBHHx and the nanocomposites were recorded to obtain insight into the nanofiller–matrix interactions (Figure 3). GO encompasses epoxide and hydroxyl functional groups on both sides of its basal plane and carboxyl moieties at the edges. The peak at ∼3500 cm^−1^ corresponds to the O-H stretching vibrations and that at about 1730 cm^−1^ is ascribed to the C-O stretching of the carboxylic acids. The band at 1620 cm^−1^ corresponds to the aromatic C-C stretching and at ∼1400 cm^−1^ corresponds to the O-H deformation and those at 1280 and 1060 cm^−1^ to the epoxy C-O stretching [28].

On the other hand, the spectrum of PHBHHx shows the C-H stretching bands in the range of 2900–3000 cm^–1^, the C=O stretching of the ester group at 1730 cm^–1^, the symmetric and antisymmetric COO^–^stretching at about 1600 cm^–^^1^ and at 1413 cm^–1,^ respectively. Moreover, C-O-C stretching vibrations appear at about 1050 and 1250 cm^–1^ [5] and the bending and stretching of the CH_2_ groups at 1150 and 1470 cm^–1^. Regarding the nanocomposites, the spectrum is fairly similar to that of neat PHBHHx, with some additional bands from the GO such as the O-H stretching that appears wider and at lower wavenumber compared to the neat polymer, ascribed to H-bonding interactions between the C=O of the ester group of PHBHHx and the OH moieties of GO. In this regard, it has been reported that the interactions between GO and molecules containing OH groups results in a widening of the O-H stretching band and causes a small downshift [29]. The same trend can be found for the carbonyl stretching band that appears at about 10 and 20 cm^–1^ lower in the nanocomposites with 1.0 and 5.0 wt% GO, respectively, compared with PHBHHx. All these facts are indicative of the strong PHBHHx–GO interactions. Similar behaviour of peak shift due to H-bond formation has been previously reported for PHB reinforced with other fillers such as ZnO [9], silica [30], pseudoboehmite [31], cellulose [32], etc. 

### 3.3. Thermal Stability 

**Figure 4:**
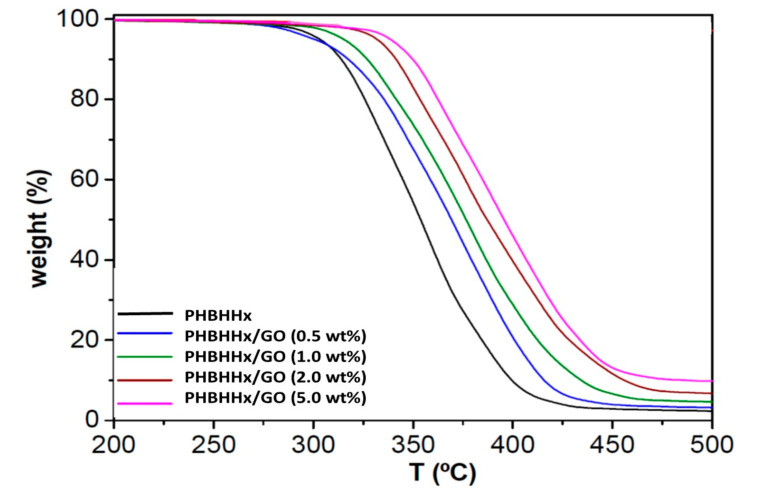
TGA curves of neat PHBHHx and the nanocomposites with different GO loading.

The thermal stability of PHBHHx and the nanocomposites was investigated under a nitrogen atmosphere and typical thermograms are compared in Figure 4. The characteristic degradation temperatures of the different nanocomposites are collected in Table 1. PHBHHx shows a single degradation stage, which is in agreement with what is reported in the literature [33] and starts (*T*_start_) at about 290 °C [34] and shows 10% weight loss (*T*_10_) at the maximum rate of weight loss (*T*_peak_) at about 314 and 325 °C, respectively. The degradation likely takes place by means of a random chain scission mechanism, resulting in smaller polymeric segments with COOH terminal groups and crotonic acid as one of the typical by-products [35]. 

A one stage decomposition process is also detected for the nanocomposites, although shifted to higher temperatures, corroborating an effect of thermal stabilisation induced by the GO. Accordingly, *T*_start_ rises by about 9, 22 and 24 °C upon the addition of 1.0, 2.0 and 5.0 wt% GO, respectively. The same tendency is observed for *T*_10_ and *T*_peak_, even with higher increments, by up to 40 °C at the highest GO loading (Table 1). These results demonstrate that the incorporation of GO increases the thermostability of PHBHHx and is likely related to its high thermal conductivity and heat absorption [36]. Thus, GO can change the dripping of polymer during the combustion process and reduces the heat release rate, hence improving the thermal stability. Further, stiff GO can strongly interact with PHBHHx, which is inferred from FT-IR analysis, by increasing the rigidity of the matrix. This restricts thermal movement of molecular segments and hence the steric hindrance increases. Therefore, in order to destroy the macromolecular structure, higher energy is required which is reflected in higher temperature. At the same time, GO evenly dispersed in the matrix can act as a barrier to gas and heat and this can also improve the thermostability of the nanocomposites [37]. On the other hand, the nanoscale size of GO deters the formation of appreciable vapor pressure and this inherently provides better thermal stability. Similar behavior of thermal stability enhancement upon GO addition has been previously reported for other polymeric matrices [25,37,38]. This improvement is desirable for high-performance food packaging applications.

### 3.4. Mechanical Performance

**Figure 5:**
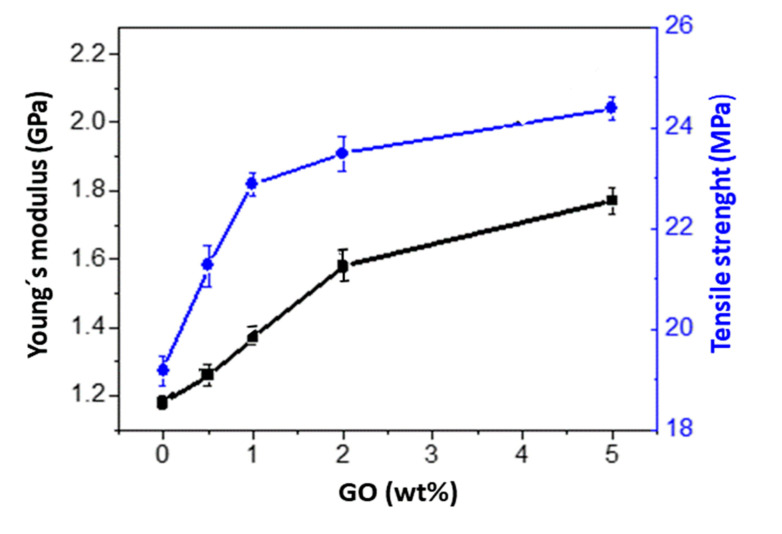
Young´s modulus and tensile strength of PHBHHx nanocomposites as a function of GO loading.

The room temperature static mechanical properties were assessed by tensile tests and the values of Young’s modulus (*E*) and tensile strength (*σ_y_*) derived from the stress-strain curves are depicted in Figure 5. Pure PHBHHx exhibits a Young’s modulus close to 1.2 GPa. The addition of GO causes a steady rise in the stiffness; the highest rise being about 40% at 5.0 wt% loading. This extraordinary increment is attributed to the strong PHBHHx–GO interfacial adhesion via hydrogen bonding interactions between the –OH groups of the GO surface and the ester groups of the biopolymer, as revealed by FT-IR analysis. Likewise, *σ_y_* rises by about 28% at 5.0 wt% loading compared to the neat polymer. This increase is higher than that reported upon the addition of other nanofillers to PHBHHx [11,39], with the additional advantage that no chemical reactions were required in our study nor the use of compatibilising agents, which renders the process environmentally friendly. Nonetheless, nanocomposites with 2.0 and 5.0 wt% GO display close σ_y_ values, indicating that the strength is levelling off and therefore higher GO loadings will not likely result in higher strength improvements. 

Taking into account the reported Young’s modulus for GO [40], the theoretical E values for the nanocomposites were estimated by the Krenchel’s rule of mixtures for discontinuous reinforcement, which can be written as indicated in the following equation:

E_c_ = (η E_f_ − E_m_) V_f_ + E_m_ (5)

where *E_c_*, *E_f_* and *E_m_* are the tensile modulus of the composite, filler and matrix, respectively; *V_f_* the filler volume fraction; and η the strengthening coefficient that is 0.2 for randomly oriented fillers. The calculated Young’s moduli of nanocomposites with GO contents ≤2.0 wt% are in very good agreement with the experimental data (differences lower than 10%). However, the data for the nanocomposite with 5.0 wt% GO is significantly lower than the predictions and this is likely due to the strong nanosheet stacking, as revealed by SEM, which limits the stress transfer ability. In fact, the model adopts perfect nanofiller dispersion and nanofiller–polymer interfacial adhesion, while the nanofillers are not individually dispersed as a monolayer but forms stacked films and shear slippage can occur within the nanosheets, which restricts the stress transfer to the matrix.

Another important issue is the material toughness, that is, the impact resistance, which can be evaluated as the area under the tensile curve [41]. Neat PHBHHx has about 25% crystallinity [7], which results in certain inherent brittleness and moderate impact resistance. It was found that the area under the tensile curve slightly decreased with increasing GO concentration, resulting in around a 11% drop at 5.0 wt% loading (Figure S3, Supplementary Materials). As is known, the state of nanofiller dispersion and its adhesion with the matrix have strong influence on the rate of energy absorption; hence, the impact properties of polymer nanocomposites [42]. Thus, the strong GO-PHBHHx adhesion via H-bonding should limit the elongation at break, that is, the ductility of the nanocomposites, which is reflected in a slightly smaller area under the tensile curve and, therefore, is a little reduced impact strength. The formation of an entangled network comprised of both composite components would render the permanent polymeric flow more difficult and this restricts the deformation of the chain segments. Overall, tensile tests reveal that the nanocomposites show a good combination of stiffness, strength and toughness. 

### 3.5. Barrier Properties

**Figure 6:**
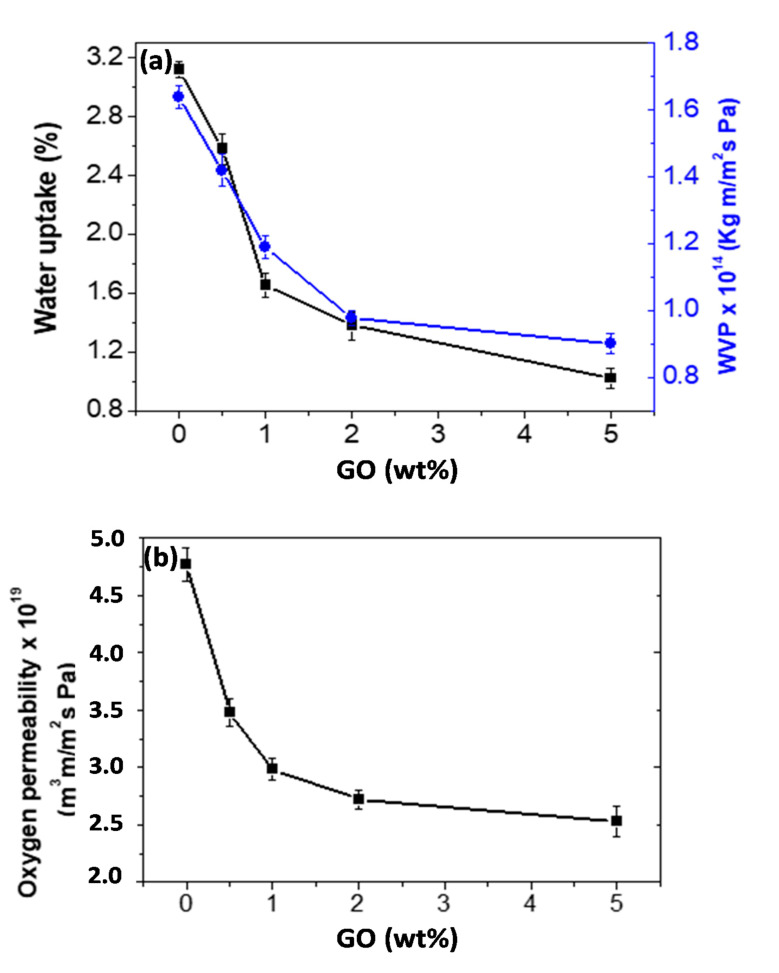
(**a**) Water uptake and water vapour permeability (WVP) and the (**b**) oxygen permeability of PHBHHx nanocomposites as a function of GO loading.

One of the main goals for adding nanofillers to PHBHHx is to improve its barrier properties for gases, vapours and organic compounds so that it can be competitive with other conventional plastics such as polypropylene (PP), polyethylene (PE) and polystyrene (PS). Water vapour and oxygen are the two most studied permeants for food packaging applications because they may transfer from the internal or external environment through the composite package wall, resulting in a continuous change in product quality and shelf-life [43]. The water uptake of the nanocomposites and their water vapour permeability (WVP), that is, the amount of water vapour that permeates per unit of area and time in a packaging material, were measured and the results are shown in Figure 6a. A strong reduction in water uptake is observed upon raising the GO concentration by about 70% at the highest loading. A similar trend is found for the WVP, which drops by about 45% at such loading. This behavior demonstrates enhanced barrier performance for the nanocomposites in comparison to the neat biopolymer and is likely associated with the presence of the GO nanosheets that is well dispersed within the matrix that increases the tortuosity of the transport path. Furthermore, due to the hydrogen bonding between GO and PHBHHx in the nanocomposites (Figure 3), a physical crosslinked network could be formed in the system where the gas penetration becomes more difficult.

Regarding the oxygen permeability of the nanocomposites (Figure 6b), a clear reduction is also observed as the GO content rises, showing a minimum (about 35% decrement as compared to neat PHBHHx) at the highest nanofiller concentration. This improved barrier performance should be related to the strong GO–polymer interfacial adhesion that causes chain immobilisation. Furthermore, due to its high aspect ratio and high electronic density of the carbon rings, GO is able to repel atoms and gas molecules and, therefore, has a very low solubility to gases [44].

Moreover, it has been demonstrated that the presence of impenetrable GO nanosheets homogeneously dispersed in the polymer matrix results in an increase in the diffusion path (tortuosity) and consequently a decrease in the gas permeability of the nanocomposites. [45]. The barrier properties of GO/polymer composites are affected strongly by the aspect ratio, dispersion and orientation of the graphene nanosheets, as well as the GO/polymer interface adhesion. The better the degree of dispersion and the stronger the interfacial adhesion, the better the barrier performance [45].

Overall, experimental data confirm that the incorporation of GO to PHBHHx matrix has a beneficial effect on the gas barrier properties, rendering improved materials for packaging oxygen-sensitive and/or moisture-sensitive products.

### 3.6. Antibacterial Properties

**Figure 7:**
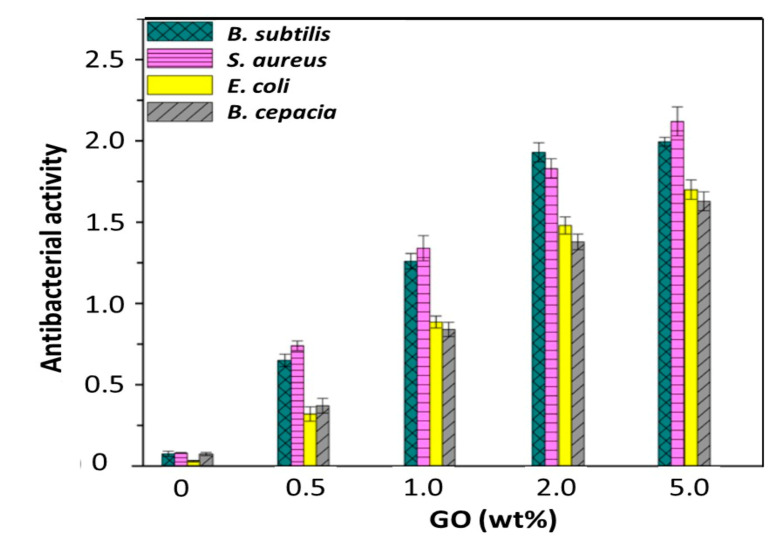
Antibacterial activity of PHBHHx and PHBHHx/GO nanocomposites against *B. subtilis*, *S. aureus*, *E. coli* and *B. cepacia*.

The antibacterial action of neat PHBHHx and the nanocomposites was explored against model pathogen bacteria: *E. coli* and *B. cepacia* (Gram negative) as well as *S. aureus* and *B. subtilis* (Gram positive) and the results are shown in Figure 7.

Neat PHBHHx hardly shows antimicrobial action, which in agreement with results reported previously [46]. In all cases, the antibacterial action grows abruptly with increasing GO concentration and the best antibacterial activity is found for the nanocomposite with 5.0 wt% GO loading. This performance can be rationalised by taking into account the very large GO surface area that allows a big interfacial contact area with the bacteria. Thoroughly, the biocide effect is stronger versus Gram positive cells, reaching a value higher than two for the nanocomposite with the highest GO content. In the same manner, effective inactivation of this type of bacteria was already reached at 2.0 wt% GO content, while for the Gram negative bacteria, the bactericide effect is moderately less effective. On the other hand, only small differences were observed between the activity against *S. aureus* and *B. subtilis* and the same applies for the inactivation of *E. coli* and *B. cepacia*, suggesting that the different toxicity is mainly related to the different characteristics of the cell wall of Gram negative and Gram positive bacteria [47]. Thus, Gram positive bacteria have a single cytoplasmic membrane and a thick wall comprising peptidoglycan layers, whereas the Gram negative have a more complex cell wall, with a layer of peptidoglycan between the outer membrane and the cytoplasmic membrane. Moreover, the differences might be connected with their different form and size.

The antibacterial activity of graphene-based materials is well known, although the mechanisms responsible for the biocide action are not fully clear yet. Several mechanisms including chemical and physical processes have been proposed [20,21]. Among the chemical mechanisms are membrane and oxidative stresses due to the generation of reactive oxygen species (ROS), which are extremely destructive to organisms at high concentrations. ROS causes peroxidation of lipids, oxidation of proteins and damage to nucleic acids as well as enzyme inhibition, ultimately resulting in the death of the cells. In particular, GO nanosheets can generate hydroxyl radicals that attack the carbonyl groups of the peptide linkages of the bacterial cell wall and damage the cellular components such as lipids, proteins and DNA, thus resulting in the destruction of the bacteria. Furthermore, the physical contact of microorganisms with GO results in the Schottky barrier formation and Fermi level alignment based on the band theory, which enables the facile transfer of electrons from bacterial membranes to graphene materials that are good electron acceptors, also destros the membrane. On the other hand, various physical mechanisms including direct contact of the GO sharp edges with the bacterial membrane, wrapping/entrapment of the bacterial cell and lipid extraction have been proposed [20]. Therefore, the Gram negative bacteria with an outer membrane are found to be more resistant to the membrane harm motivated by the GO nanosheets than the Gram-positive one that lacks the external membrane.

Overall, experimental results reveal the great potential of the nanocomposites with high GO loading to inhibit the growth of human pathogenic bacteria.

## 4. Conclusions

In this study, nanocomposites made of biocompatible and biodegradable PHBHHx copolymer and different GO concentrations have been prepared via a simple, cheap and sustainable method of ultrasonication followed by solution casting. Their morphology, crystalline structure, thermal, mechanical and antibacterial performances have been investigated. Outstanding improvements in mechanical properties (about 40% in stiffness), thermal stability (up to 24 and 40 °C in the start and peak degradation temperatures, respectively) and barrier properties (up to 45 and 35% drop in water vapour and oxygen permeability) were found. These are ascribed to the strong interaction between GO and the biopolymer via hydrogen bonding, as demonstrated by FT-IR analysis. More importantly, these novel nanocomposites show strong biocide action against Gram positive *S. aureus* and *B. subtilis*, as well as Gram negative *E. coli* and *B. cepacia* and the antimicrobial activity increases with increasing GO concentration. PHBHHx/GO nanocomposites, in particular with 5.0 wt% GO loading, are an interesting alternative to petroleum-based polymeric materials for use in a variety of food packaging applications.

## Figures and Tables

**Table 1:** Roughness and thermal stability data of PHBHHx/GO nanocomposites.

Sample	R_a_ (µm)	*T*_start_ (°C)	*T*_10_ (°C)	*T*_peak_ (°C)	R (wt%)
PHBHHx	0.22	280	314	325	2.2
PHBHHx/GO (0.5 wt%)	0.25	277	316	339	2.9
PHBHHx/GO (1.0 wt%)	0.36	289	325	343	4.0
PHBHHx/GO (2.0 wt%)	0.59	302	343	361	6.1
PHBHHx/GO (5.0 wt%)	0.78	304	348	365	10.7

R**_a_**: mean surface roughness; *T*_start_: temperature at which the sample starts to lose mass; *T*_10_: temperature at which 10% of the mass are lost. *T*_peak_: temperature of maximum rate of weight loss corresponding to the peak of the derivative. R: residue at 500 °C.

## Data Availability

The data supporting the findings of this study are available within the article and its supplementary materials.

## Supplementary Materials

The following are available online at https://www.mdpi.com/article/10.3390/polym13142233/s1, Figure S1: Photographs of neat PHBHHx and PHBHHx/GO (5.0 wt%), Figure S2: Histograms of the thickness distribution obtained from GO nanosheets, Figure S3: Area under the tensile curve as a function of GO loading.
