# Supplementary material for: Effect of Graphene Oxide on the Properties of Poly(3-Hydroxybutyrate-co-3-Hydroxyhexanoate)
Source: Polymers (Basel). 2021 Jul 7;13(14):2233. doi: 10.3390/polym13142233 (PMC8309387; doi:10.3390/polym13142233)
Supplement: Supplementary file 1 [file polymers-13-02233-s001.zip › polymers-1278007-supplementary.pdf]

## Supplementary Materials

# Effect of Graphene Oxide on the Properties of Poly(3-hydroxybutyrate-*co*-3-hydroxyhexanoate)

**Ana M. Díez-Pascual** *

Universidad de Alcalá, Facultad de Ciencias, Departamento de Química Analítica, Química Física e Ingeniería Química, Ctra. Madrid-Barcelona, Km. 33.6, 28805 Alcalá de Henares, Madrid, España (Spain);

* Correspondence: am.diez@uah.es; Tel.: +34-918-856-430

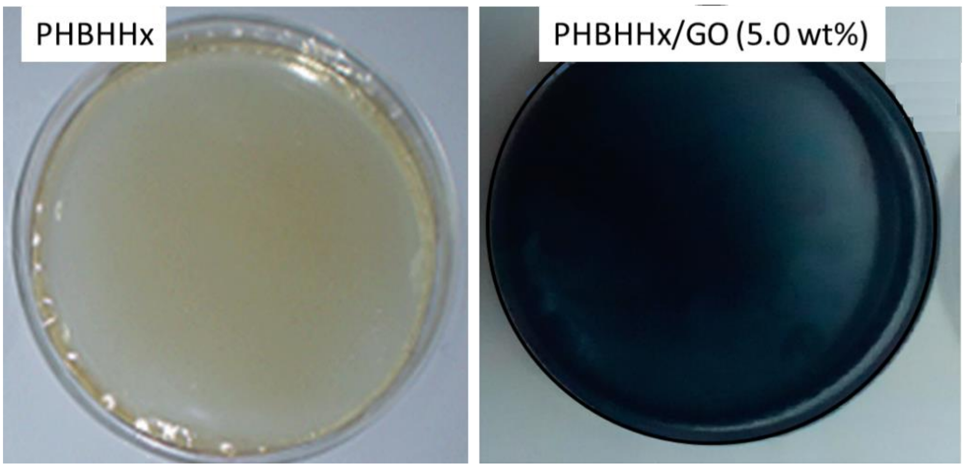


**Figure S1.** Photographs of neat PHBHHx and PHBHHx/GO (5.0 wt%).

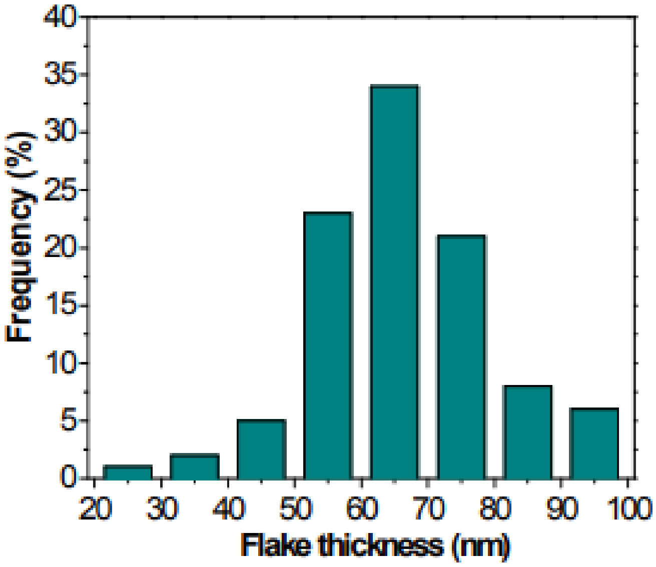


**Figure S2.** Histograms of the thickness distribution obtained from GO nanosheets.

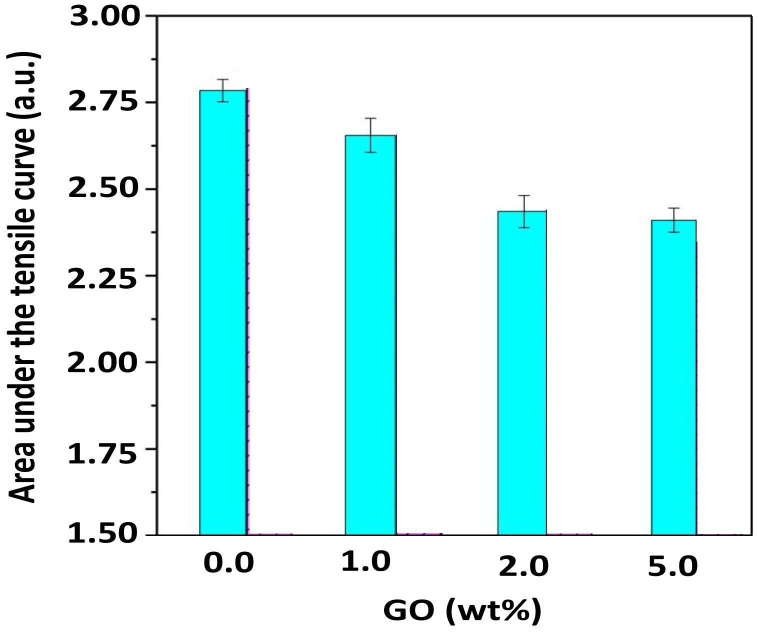


**Figure S3**. Area under the tensile curve as a function of GO loading.
